# A qualitative exploration of the lived experiences of Body Dysmorphic Disorder

**DOI:** 10.3389/fpsyt.2023.1214803

**Published:** 2023-10-03

**Authors:** Sarah N. Brennan, Susan L. Rossell, Imogen Rehm, Neil Thomas, David J. Castle

**Affiliations:** ^1^Centre for Mental Health, Swinburne University of Technology, Melbourne, VIC, Australia; ^2^Gatehouse Centre, Royal Children’s Hospital, Melbourne, VIC, Australia; ^3^Department of Psychiatry, St. Vincent’s Hospital, Melbourne, VIC, Australia; ^4^Institute of Health and Sport, Victoria University, Melbourne, VIC, Australia; ^5^Department of Psychiatry, University of Tasmania, Hobart, TAS, Australia

**Keywords:** Body Dysmorhic Disorder, lived experience, intolerance of uncertainty, qualitative research, the flawed self, consumed by the disorder, interpretative phenomenological analysis

## Abstract

Body Dysmorphic Disorder (BDD) is characterized by an intense preoccupation with one or more perceived “defects” in physical appearance. Despite the distress and impairment associated with BDD, the disorder remains understudied and poorly understood. In particular, there are limited studies available which give voice to those with firsthand experiences of the disorder. A qualitative approach was employed to study lived experience of BDD. In-depth semi-structured interviews were conducted with 12 participants with BDD, aiming to understand their subjective experiences of the disorder. Data was analyzed using Interpretative Phenomenological Analysis (IPA). The results identified three superordinate themes; (1) consumed by the disorder, (2) the flawed self, and (3) intolerance of uncertainty about appearance. The qualitative findings of this study are discussed in relation to current conceptual understandings of BDD, including the cognitive behavioral model.

## Introduction

Body Dysmorphic Disorder (BDD) is a psychological condition characterized by an intense preoccupation with one or more perceived defects in physical appearance, repetitive appearance-related behaviors (e.g., mirror checking, reassurance seeking, skin picking) or pervasive mental acts (e.g., comparing self-appearance to others), and is marked by clinical levels of distress and functional impairment (1). The Diagnostic and Statistical Manual of Mental Disorder (DSM-V) classifies BDD as an Obsessive-Compulsive Related Disorder [(1); OCDR]. BDD has been conceptualized as an OCRD due to the symptom overlap, high comorbidity, and neuropsychological similarities between BDD and OCD (2). BDD, however, is considered a distinct condition as the persistent preoccupations (obsessive-like thoughts) and repetitive acts (compulsion-like behaviors) seen in BDD are solely focused on appearance-based concerns.

Large epidemiological studies reveal BDD is relatively common affecting approximately 1.7–2.9% of the general population (3–5). BDD tends to affect males and females in similar proportions and typically emerges during adolescence, with a mean age of onset of 16.4 years (6). Despite its high prevalence and early onset, accurate clinical diagnosis and in turn access to appropriate treatments is delayed several years, contributing to a chronic and debilitating course trajectory (7).

The cognitive behavioral model of BDD proposes that a crucial factor to the development and continuation of the disorder is a process of selective attention [e.g., (8–12)]. This model postulates that a vicious cycle commences when an individual experiences either an external (e.g., seeing image of self in the mirror) or internal (e.g., a ‘felt’ impression of the body part) trigger, which activates a process of self-focus attention whereby the individual selectively attends to specific aspects of their appearance as opposed to the ‘bigger picture’ and attaches negative appraisals and maladaptive interpretations to this experience. Although described as a discrete process it is suggested that this process can often be perpetually ‘turned on’ for these individuals further strengthen this cycle. Veale (8) refers to this process as “self as an esthetic object” and suggests that individuals are consumed by imagery or “felt impressions” of their appearance such that they experience themselves from an observer perspective.

Research, to date, has primarily endeavored to understand the clinical symptoms and underlying constructs of BDD predominantly using quantitative methods. In recent years, there has been a small number of qualitative studies which have approached individuals with BDD to ask about their first-hand experiences. Research of this nature is paramount to evaluating current theoretical models and extending upon gaps in current knowledge. Silver and colleagues interviewed 11 individuals with BDD using participant’s self-photographs and used a narrative analysis to understand the way in which they perceived their appearance (13, 14). Identified themes included increased threat perception resulting in disordered interpersonal relationships; a wish for regularity and symmetry in physical appearance; idealization of childhood self; a sense of duty to look good; and a focus on specific details rather than on ugliness. In a follow-up study (15), mirror gazing was explored in 10 individuals with BDD. Participants described mirrors as controlling, imprisoning, and disempowering forces that had a crippling and paralyzing impact on their lives. A further study of eight individuals with BDD used inductive thematic analysis and identified three core themes; routine and repetitive, safety through control, and natural and automatic. Appearance behaviors in BDD were complex and did not appear to follow a straightforward model of reward and punishment, such that some behaviors (such as camouflaging through the use of make-up) provided a sense of relief and reassurance, whereas others (such as mirror checking) could be highly distressing. There was thus a paradoxical pattern, whereby participants were seemingly dissatisfied with BDD behaviors, yet also derived comfort, reassurance and a sense of identity from them (16).

A Swedish study explored experiences of the health care system among people with BDD (17). Six themes emerged; being absorbed in time-consuming procedures, facing tensions between one’s own ideal and perceived reality, a sense of becoming the disorder, feeling restricted in one’s life, attempting to reduce one’s problem’s (through avoidance and safety behaviors), and striving to receive care but encountering difficulties with the health care system. Challenges in attempting to access health care included feeling that they were not being taken seriously, a lack of knowledge specific to BDD among health professionals, and a scarcity of referral options.

Finally, a recent qualitative study interviewed 8 individuals who self-identified as living with BDD (18). The study endeavored to understand the developmental origins individuals with BDD attributed their experience to. Four themes were identified: exposure to bullying and external critique of appearance; experiencing rejection, shame, and sense of not being enough; developing an awareness of the solidification of concerns; and learning about and reflecting on triggers. This study called for further qualitative studies to amplify the voices of those living with this condition and better inform conceptual models of BDD.

Interpretative Phenomenological Analysis (IPA) is a qualitative research method (19) that goes beyond thematic descriptions to create a rich analysis of how a sample of individuals perceive, appraise, and “make sense” of their experiences (20, 21). IPA assumes that individuals are self-reflective beings who are not only capable of, but actively seek to, engage in meaningful interpretation of their life experiences. IPA promotes a method of double hermeneutics. Firstly, the participant is making sense of their world, and secondly, the researcher works to decode that meaning (21).

This study employed IPA to examine the lived experiences of individuals with BDD. In line with IPA guidelines this study did not endeavor to test hypothesis but rather to address two broad research questions:

1. What are the subjective experiences of living with BDD and its impacts?
2. How do these experiences fit with current theoretical understandings of BDD?

## Materials and methods

### Design

A qualitative research design, using IPA (20, 21) was employed. The project was approved by Swinburne University and St Vincent’s Hospitals’ Human Research Ethics Committees and informed consent was obtained from all participants.

### Participants and procedure

Twelve people with BDD (7 female) were recruited via two specialist BDD services. Participants were eligible if they were aged ≥18 years, were proficient in spoken and written English, and had a current and primary diagnosis of BDD according to DSM-IV criteria. Participants were ineligible if they had a neurological disorder, current alcohol or drug abuse requiring clinical attention, and a current or lifetime psychotic disorder. Participants with other psychological comorbidities were included, to support generalizability. Interviews were between 60- and 90-min duration and conducted in-person at a hospital or university mental health research center. The female interviewer (SB) was a clinical psychology doctoral student at the time of interviewing and had no pre-existing relationship with the participants.

### Measures

The following measures were administered to participants to characterize the sample. The *MINI International Neuropsychiatric Interview* [MINI 6.0; (22, 23)] and the *Body Dysmorphic Disorder Diagnostic Module* [BDD-DM; (24)] confirmed DSM-IV diagnosis and comorbidities. The *Yale-Brown Obsessive Compulsive Scale Modified for BDD* [BDD-YBOCS; (25)] is a 12-item semi-structured clinician administered interview that assesses BDD symptom severity during the past week. The BDD-YBOCS produces subscale scores for Obsessions (range 0–20), Compulsions (range 0–20) and Insight/Avoidance (range 0–8), as well as a total symptom severity (range 0 to 48). The *Brown Assessment of Beliefs Scale* [BABS; (26)] is a 7-item clinician-rated scale that measures the degree of conviction and insight associated with a primary obsession or delusional belief over the past week. The *Depression Anxiety Stress Scales-21* [DASS-21; (27)] is a 21-item self-report scale comprised of three subscales: depression, anxiety, and stress.

A *Semi-Structured Qualitative Interview* explored participants’ subjective experience of living with BDD. A small set of standard questions and prompts were developed by authors (SB, SR, and NT) which enquired about (1) onset and early course of BDD; (2) thoughts, emotions, and behaviors associated with BDD; (3) impact of BDD on every-day life; and (4) participant’s reflections on the causes and function of their experiences. The technique of ‘funneling’ was used such that the interviewer asked questions about broad topics first to allow the respondent to reflect general views followed by prompts to direct the participant to more specific content. Each interview commenced with the statement and question of *“I am interested in understanding your personal experiences of Body Dysmorphic Disorder and the meaning you attribute to experiencing these appearance-based concerns. Could you start by telling me about what Body Dysmorphic Disorder has been like for you?.”* Participants were encouraged to consider their beliefs, explanations, and the meaning they attributed to each aspect of their BDD experience.

### Data analysis

All qualitative interviews were audio recorded, manually transcribed verbatim, and analyzed according to IPA methodology (20, 21). A detailed analysis of each participant’s interview occurred before moving onto the next participant. The author who conducted the interviews (SB) listened to and re-read the interview transcript several times, while making written annotations. The next step involved developing concise phrases, compatible with theoretical concepts and psychological terminology (i.e., interpretation), to develop a list of initial themes. Next, themes were clustered by looking for connections and a sense of order, such that emerging superordinate (higher order) and subordinate (lower order) themes were developed for the individual participant with reference to key quotations from the source material. The 12 participants’ theme tables were then compared and contrasted. Factors such as richness of passages, level of meaning held by the participants, and prevalence (to an extent) were used to guide the decisions about final themes. Following credibility guidelines for qualitative research (28), clean interview transcripts and the corresponding tables of superordinate and subordinate themes were checked by a second author (IR; a clinical psychology academic with experience in IPA). Themes were discarded if they were not supported by rich evidence, had low prevalence within transcripts, or could be subsumed under other themes. Development of themes were periodically reviewed and discussed with a further researcher (NT; a senior clinical psychology academic).

## Results

The 12 BDD participants’ ages ranged from 19 to 64 years. Participants self-reported a duration of illness ranging between 6 months to 48 years (*M* = 16.23, SD = 14.08); with BDD onset at a mean of 21.75 years old (SD = 12.24). Majority of participants (75%) were currently employed on a fulltime basis; 75% were currently single; and 92% were Australian born. The participants were highly educated with an average total number of years of education of 16.54 (SD = 3.83), and the majority holding higher education qualifications.

Consistent with other BDD samples [e.g., (16)], the majority of the participants (9 out of 12) had at least one other psychiatric condition with the total number of comorbidities per participant ranging between 0 to 3. Comorbidities included Major Depressive Disorder (58%), General Anxiety Disorder (58%), Obsessive Compulsive Disorder (17%) and Trichotillomania (8%). Seven participants (58%) were currently being treated with psychotropic medication, most commonly antidepressants. On average, the participants were preoccupied with 3 body parts of concern, with a range of 1 to 5 (see Table 1). The most common concerns related to skin complexion (e.g., acne, scars, skin conditions, freckles, moles), hair (e.g., head hair loss, excessive or too dark body hair), and facial features (e.g., the shape or size of the nose, eyes, or lips).

**Table 1:** A summary of BDD appearance concerns.

Body part of concern	Number of participants endorsing
Skin Complexion	5 (42%)
Hair	5 (42%)
Head	1 (8%)
Face	4 (33%)
Nose	3 (25%)
Eyes	2 (17%)
Eyebrows	1 (8%)
Teeth	2 (17%)
Mouth	1 (8%)
Cheeks	1 (8%)
Ears	1 (8%)
Lips	1 (8%)
Jaw	1 (8%)
Chin	3 (25%)
Neck	1 (8%)
Breasts	2 (17%)
Genitals	1 (8%)
Body Frame/Body Symmetry	1 (8%)
Body Weight/Body Shape^b^	3 (25%)

^a^No participant was excessively concerned with body shape or weight alone. ^b^As participants experienced multiple areas of concerns the total number of participants experiencing these concerns exceeds the total number of participants. All participants with multiple areas of concern were however able to identify their most predominant one.

The BDD-YBOCS scores showed that average BDD symptom severity was ‘Moderate–Severe’; total score (M = 23.42, SD = 6.64, Range = 14–36), obsession subscale (M = 11.08, SD = 3.03, Range = 7–16), compulsion subscale (M = 9.08, SD = 1.68, Range = 1–15) and insight/avoidance subscale (M = 3.08 SD = 1.68, Range = 0–7). The average BABS score (M = 11.27, SD = 3.93, Range = 7–19) classified the sample overall as having ‘fair’ insight into BDD beliefs. Only one participant met Eisen et al.’ (26) criteria for ‘delusional’ conviction associated with their BDD belief (a total score of ≥18 and a score of 4 on item 1, which relates to conviction). The DASS-21 scores for depression were (M = 6.17, SD = 4.17, Range = 2–11), anxiety (M = 3.17, SD = 1.08, Range = 2–5), and stress (M = 6.25 SD = 2.69, Range = 2–9).

The IPA analysis identified three master themes, each reflecting core subjective experiences of living with BDD. The master (superordinate) themes, secondary (subordinate) themes, and example quotes are presented in Table 2.

**Table 2:** Superordinate and subordinate themes of the interpretative phenomenological analysis.

Superordinate themes	Subordinate themes	Examples
Consumed by the disorder	Controlled by one’s thoughts and behaviors	*I do not even know how they crept back in. But within one week it was back to googling procedures, taking time off work, and just not being able to concentrate on anything else […] When it was really bad I could not drag myself away from the mirror. I would be standing there staring at it for hours, I could not stop, I could not drag myself away because I needed to be looking at it* (Participant 1). *It was the only thought I could think about 24/7. I would go to the mirror 50 times per day. I could not sit still and I just kept checking the mirror, taking videos of myself, photographs on my phone, inside the house, outside the house, in different rooms, different mirrors, different lighting, asking my family, asking my friends* (Participant 2). *It was this obsessiveness of looking at others and then just these constant thoughts over and over, nearly all day long […] I was constantly feeling it, constantly going to the mirror, thinking ‘does it look okay here?’, and then trying to avoid the mirror. But then I look at other reflections or look at photos close up and it was just constant […]It was the thoughts, it’s what I did with it, and everything else involved. It has dominated my life so much*. (Participant 3).
	Trapped within one’s body	*I felt ashamed and anxious that I would have to live in my body for the rest of my life, that I wasn’t good enough and that I wasn’t lovable […] This complete anxiety that I would have to live in this body for the rest of my life; disgusted by the way I look.* (Participant 4). I feel like I’m trapped in the wrong person’s* body […] because my body just will not change (Participant 5).
	Hopelessness and ruminating about death as an escape	*I just could not enjoy anything. I just stopped enjoying the things I used to enjoy. I just could not concentrate on them because they felt insignificant compared to this […]. I sort of take comfort in knowing that it’s not going to last forever. That I am going to die one day, so you know, even if it does last my entire life it’s going to be over some day.* (Participant 2). *At its worst, I have not wanted to go on anymore, when feeling it is always going to be like this; it is exhausting […]. I have even thought I just wanted to cut it off completely or that I’d rather not be alive and living with these emotions. I have thought I wish I could just go to bed and not get back up again* (Participant 3).
	Lost opportunities and impact on relationships	*BDD is very debilitating; it holds you back from so many things. I feel I could have been and done so many things […]. I have ruined a lot of friendships because of this* (Participant 6). *I could have done things. I could have got married* (Participant 7). *It has created a barrier between me and other people. It is particularly prominent in relation to romantic endeavors* (Participant 8).
The flawed self	External flaw as a symbol of one’s inner flawed self	*I had this bolt out of the blue that I was this bad person, like undeserving and bad and therefore ugly, you know. And for some reason it had to do with my X* (Participant 9). *I believe that if people were looking at me, they would think I’m ugly. Like, that’s the first thing they would think ‘ugly’, and that would somehow also correlate with me being a bad person* (Participant 10).
	Self as fundamentally abnormal	*I just feel different to others […] Just feeling there is something not right. I feel inferior to others in a sense, and that is what I have always felt* (Participant 3). *I think who would want to be with someone like me. Because I am just not, I just do not feel like normal* (Participant 5). *I went out at night once and saw my reflection in a window. I just thought how could I do that to the world, the planet, how could I go out and inflict this on them? I am like this revolting thing […]so I went back home* (Participant 11).
	Objectified and exposed self	*I felt completely on display to the public* (Participant 6). *I remember just driving for the first time, I felt so exposed, so aware of myself […]. Just knowing that people could see me when I was out in public* (Participant 4). *When I am not happy with how I’m feeling or how my body is feeling, I literally can feel the clothes on me and how they feel against my body parts. I’m constantly aware of it* (Participant 12). *It’s not just what it looks like, I can also feel my X beings small* (Participant 10).
Intolerance of uncertainty about appearance	Doubt and uncertainty	*A thought would always come into my mind saying ‘maybe you missed something’…That feeling of dread. That even if it has not changed for a month, it’s not guarantee that it will not change tomorrow. So, I am always dreading it* (Participant 2). *It was awful. Thinking I have seen something and then going back and thinking ‘hang on maybe I have not, maybe I imagine it’. Almost like trying to catch myself. Arranging my X in a way to think ‘hang on yes there it is’* (Participant 9).
	Just not right	*I became concerned my X was asymmetrical. It has to do with symmetry. I think even when I was quite young, I was concerned with symmetry* (Participant 9). *Sometimes I cannot go out because I’ve just been – it’s just been ruined, I cannot find that right feeling- that feeling I am after* (Participant 12). *Sometimes I see the mirror and things are going good, but then I go out and I will think how did I miss that. So, then it’s like how can I capture that feeling again? I’ll capture it and then something will happen and it’s completely the* opposite (Participant 5). *I’ll look really up close at it and if I could just find the time where it does not look so bad then it can give me a bit of that feeling […] It’s ‘oh hang on, it does not look too bad’, and then you walk away with it. So, it’s trying to find that feeling.* (Participant 3).
	Focus on detail over the whole	*I get close up to the mirror […] I get up really close and I start scrutinizing each area and then I’ll pick or scratch it. I would scan everything for imperfections one bit at a time* (Participant 1). *I’ll zone into that area […] I can actually like feel that part of my body. Then you almost zone into that area on your body and you can feel it just being. It’s, it’s weird. I guess it is a heightened sense of, you know, sensation and things* (Participant 12).
	Seeking certainty and control through confirmation	*I was terrified that there was something visible but at the same time I wanted there to be. It sounds like madness, but it was actually a relief. It was a bit like I wanted the confirmation but at the same time I dreaded it. I was torn between not wanting it to be and needing it to be there. It was a terrible bind […] It gave me something definite rather than the uncertainty. It needed certainty. When I have been unwell what I could not cope with was the uncertainty* (Participant 9). *I keep photos. It’s like I want the evidence to say it is there, you are not making it up. There is a flaw there, even though it might be only be tiny, it’s that reassurance […] Sometimes I have to go check that it is there. I’ve got to put my finger over it to feel the unevenness or look at it in the mirror a little bit to make sure that it is there, to know it is there. And then I have felt this sense of, uh, like a bit of a release* (Participant 3).
		*It gave me a sense of control and a nice sense of isolation and there was no worry because I did not have to think about the future or jobs or any really big scary things that were out of my control[…] When I am obsessed with my BDD the control that I can get is I can live in my own little world where the only thing that matters is this X and the only thing, I need to do in order to have a successful fulfilling life is to get it fixed*” (Participant 1).

Reference to specific body parts of concern have been replaced with the symbol X to protect the anonymity of participants. The symbol * has been used to represent a word replacement used by authors to exclude potentially identifiable data.

### Consumed by the disorder

This master theme highlights the all-consuming nature of BDD symptoms, the discomfort and distress associated with one’s physical embodiment, and the functional impact of these experiences. This is explained further within four sub-themes.

#### Controlled by one’s thoughts and behaviors

Participants described feeling controlled and tormented by obsessive thoughts relating to their body part of concern and associated compulsive behaviors. Some referred to “the BDD” as a distinct entity that had the capacity to hijack or dominate them. In exploring the purpose of BDD behaviors, the participants identified two core functions, one being an attempt to improve or hide the body part of concern, and the other stemming from a strong desire “to know the truth” regarding their appearance; that is, to gather evidence to confirm or disprove their “deformed,” “ugly,” or “unacceptable” appearance. Participants reported that the latter ‘investigative’ type of behaviors were associated with higher distress, stronger urges, and that these behaviors often felt illogical.

Participants reported finding themselves stuck in checking routines and feeling compelled to complete every possible option associated with the behavior, such as changing the location, lighting, angle, or the mirror used. They referenced being driven by the need to know exactly how they looked but were often left feeling unsure, not knowing which of the images or impressions they should trust, that is, which represented their ‘true’ appearance.

The mirror played a central role for many, along with several other visual and tactile methods used to perform checking behaviors. These included taking photographs and videos, with some participants storing files on electronic devises and using zoom and editing functions to evaluate the body part. Some kept diaries including pictures, descriptions, and measurements of the body part(s) of concern.

#### Trapped within one’s body

Participants described the sense of being trapped within their body, with a sense of discomfort with their physical embodiment. They felt their body was “not right” or even fundamentally wrong.

#### Hopelessness and ruminating about death as an escape

All participants identified experiencing a strong sense of hopelessness and futility as a result of their BDD experiences. They described an inability to experience pleasure or happiness during their most challenging periods; over half had contemplated suicide or had made a previous suicide attempt.

#### Lost opportunities and impact on relationships

Participants reported that BDD infiltrated most aspects of their lives including work, studies, relationships, and physical intimacy. Many expressed feeling held back in life as a consequence of BDD and unable to pursue their goals and aspirations. Participants expressed guilt and shame associated with believing they were a burden to their partners, family, and friends.

### The flawed self

Participants invariably viewed themselves as being fundamentally flawed. They felt acutely self-conscious, had a heightened awareness of their physical body, and were fearful of being seen by others, resulting in them feeling vulnerable, exposed, and wanting to hide from the world. The flawed self was expressed in three ways.

#### External flaw as a symbol of one’s inner flawed self

This theme was so strongly embedded in participants’ accounts that they spoke about the qualities of their physical body part and their inner self as if they were one and the same thing. They suggested that people would somehow “know” or be able to “see” inner negative personal qualities such as weakness, inadequacy, inferiority, or badness by simply viewing their appearance concern. It was as though they believed that if others were able to detect the physical flaw, this would somehow be a confirmation of an inner flawed being.

#### Self as fundamentally abnormal

Participants viewed themselves as fundamentally abnormal, with a strong associated sense of shame and guilt. They did not simply view themselves as imperfect, less attractive than they aspired to be, or as failing to reach a beauty standard, but rather experienced themselves as inherently defective, abnormal, and ‘wrong’. Many of the participants reflected that these feelings predated BDD symptom onset.

#### Objectified and exposed self

Participants described hyper-awareness of their physical body, especially when they were in public settings. They reported feeling like objects which were on display and being evaluated, judged, and ridiculed by others. Many reported visualizing their body or body parts from an observer perspective. Some participants responded by contorting or positioning their bodies in various ways as though sculpting an object which may be better received by others. For a number of participants their experience of intense physiological awareness was so strong that they reported being able to “feel” their body part on a sensory level.

### Intolerance of uncertainty about appearance

The third master theme captures experiences of incessant doubt, ambivalence, and intolerance toward uncertainty about appearance and “not just right” experiences. Four subordinate themes were identified.

#### Doubt and uncertainty

A strong experience, which emerged for all participants, was a sense of doubt, uncertainty and ambiguity regarding their perceptions and the true nature of their body parts of concern. Participants reported being stuck and struggling to move forward with daily tasks or life in general in the absence of absolute clarity and certainty about their appearance. Thus, BDD behaviors were often carried out with a goal to investigate and gain a sense of certitude regarding appearance and in turn ease the distress associated with this uncertainty. They described persistent intrusive thoughts driving this sense of doubt and uncertainty; “*How does it look right now?,” “What if it has changed?,” “Has it worsened/improved?”* and most frequently “*What if I missed something?.”* Several participants articulated that they were more distressed by the enduring uncertainty surrounding the reality and/or existence of the appearance concern than the actual existence of a physical flaw itself. Participants experienced conflicting perceptual information and an inability to trust their perceptual experiences. Although only a few participants believed their body part actually “changed” day to day, many struggled with the concept that it could change at any moment and agonized about not knowing exactly how it looked at any given moment.

#### Not just right

Connected with the theme of intolerance of uncertainty about appearance were phenomena of “not just right” experiences, coupled with a sense of unease and an urge to perform investigative compulsive acts or to engage in avoidance. Participants additionally expressed craving and longing for a “just-right” feeling. These impressions of rightness or wrongness appeared to be fuelled by felt impressions or ambiguous sensory and perceptual feedback, which the participants relied upon to make decisions, such as whether they could leave their home or when a checking behavior was complete and could be ceased.

#### Focus on detail over the whole

Participants described engaging with their body and their visual image in a detailed and piecemeal manner, repeatedly referring to practices of “zooming in” or “looking up close” at specific body areas, seemingly lacking a more holistic impression of their body/appearance. This focus on detail was further evident in the way participants listed each specific body part as if it was a fragmented entity disconnected from an overall body. This detailed way of engaging with their body appeared to drive “not just right” experiences through a process of selective attention and hypersensitivity to ambiguous perceptual and sensory bodily feedback.

#### Seeking certainty and control through confirmation

Experiences of incessant doubt, uncertainty, and “not just right” experiences were associated with high levels of discomfort and distress. In response, participants engaged in compulsive checking, trying to establish certainty and control. A number of participants shared that at times they wanted to establish evidence that they in fact looked “okay”; but in not being able to reach this conclusion they would then turn to search for evidence of the existence and abnormality of their perceived flaw, which in itself could provide some sense of relief and comfort. One participant described this as a “terrible bind” in which they found themself both desperately longing to find the flaw and dreading this confirmation at the same time. Another shared that finding evidence of the flaw not only provide a sense of relief but could at times also foster a sense of hope, as this meant that something could be done to ‘fix’ the problem. Finally, one participant shared that they found a sense of control through their preoccupation with their body part of concern as it meant they could avoid bigger life decisions and their uncertainty around the future.

## Discussion

This study aimed to build upon existing research by using IPA to examine the lived experience of BDD. Specifically, it endeavored to understand subjective experiences of those living with BDD and the ways in which these experienced impacted on those living with this condition. It additionally aimed to explore how these subjective experiences fit within current theoretical models of BD. The analysis identified three superordinate themes; (1) consumed by the disorder, (2) the flawed self, and (3) intolerance of uncertainty about appearance. Each of these themes is discussed with reference to the research literature.

*Consumed by the disorder*, summarized the subjective experiences of BDD symptomology, reflected some of the most challenging aspects of the condition and how these experiences impact upon the individual and their lives. Participants identified feeling controlled by obsessions and compulsions, feeling trapped in their bodies, and experiencing feelings of hopelessness and a desire to escape themselves. These experiences were reported to significantly impact upon daily living including employment, education, and social functioning. High levels of depression, suicidal ideation and past suicidal behaviors were identified, corresponding with clinical descriptions in the literature and highlighting the significance of suicidality as a major clinical concern for this population (6, 29, 30).

Overall, participants’ personal accounts of intrusive appearance-related thoughts and repetitive behaviors captured the obsessive and compulsive nature of BDD. The participants described doubt-based intrusions (i.e., “have I missed something?”), preoccupations with symmetry, “not just right” experiences and use of compulsive checking behaviors to manage uncertainty. The participants identified repetitive behaviors such as checking their body part of concern, comparison with others and seeking reassurance from others as central BDD symptoms. They identified a “need” to know exactly how they looked as a driver of the checking behaviors, albeit they acknowledged that checking often did not make them feel better, could worsen their distress, or at best provided only brief relief. These findings are consonant with those of Veale and Riley (31) who, in a retrospective forced-choice questionnaire, found that individuals with BDD, in contrast to controls, were motivated to check mirrors for three primary reasons; they (1) hoped that they may look different, (2) believed that they would feel worse if they did not check, and (3) desired to know exactly how they looked. Further, Windheim et al. (32) found that people with BDD were distressed both before and after mirror-gazing sessions. Baldock, Anson, and Veale (33) suggested that mirror-checking in BDD may persist despite distress, as individuals with BDD are more likely to use internal goals (e.g., needing to feel “right” about their appearance) compared to control participants who tended to have external goals (e.g., having finished applying makeup), and that in BDD these ambiguous internal goals were relied upon to inform their stop-criteria for mirror-use. Supporting this, the accounts of our participants suggest use of internal goals, for example, pursuing the “just right” feeling. The participants also provided examples of using ambiguous internal feelings to guide decisions, such as whether they could disengage from compulsive behaviors or whether they felt acceptable enough to leave the house. Further research should endeavor to understand better, the nature of distress and relief experiences associated with various BDD behaviors, as this could inform treatment paradigms.

The current results support assertions from previous qualitative research that BDD behaviors might be best understood if differentiated into categories based on their function (9, 16). The current diagnostic criteria for BDD refer to these behaviors as “repetitive behaviors,” thus avoiding the term “compulsions” although they evidently do parallel compulsions as seen in OCD. In OCD, compulsions are differentiated from safety behaviors such that safety behaviors are aimed at avoiding adverse experiences whereas compulsions are an attempt to undo or neutralize uncomfortable thoughts and/or feelings (34). Our participants described classic safety behaviors (e.g., camouflaging through makeup, or hiding under hair or clothing), which were not associated with the same level of distress as accompanied compulsive behaviors (e.g., checking behaviors, comparing oneself to others, reassurance seeking) arguably because the former are driven by a more explicit, external, and thus attainable goal and the individual believes the flaw to exist and that their appearance is being improved or protected by their actions. This is opposed to compulsive behaviors, which appear driven by a search for a sense of certainty, resulting from a core intolerance of the unknown and ambiguity. Perhaps then, these checking behaviors persist because this uncertainty is so insufferable that even a small opportunity to neutralize these feelings and gain a sense of control, even if only temporarily, is enough to reinforce them. The qualitative results of this study suggest that the distinction of ‘safety behaviors’ and ‘compulsive behaviors’ as used in OCD is relevant for BDD.

The second master theme was *the flawed self*. Participants invariably viewed themselves as fundamentally flawed. These beliefs went beyond a concern of imperfection, but of viewing themselves as wholly defective, abnormal, and wrong. Participants described self-consciousness and a hyper-awareness of their physical body including strong sensory feedback, resulting in them feeling vulnerable, exposed, and wanting to hide from the world. This master theme supports previous qualitative research, including that of Brohede et al. (17) who identified feelings of abnormality and a longing to be normal. The subtheme, *objectified and exposed-self*, strongly resonates with Veale’s (8) cognitive-behavioral model of “self as an aesthetic model,” which proposes that BDD is marked by extreme self-consciousness and self-focused attention, leading to a focus on felt impressions and engagement with mental imagery with strong sensory qualities, fuelling a selective-attention bias cycle (8).

The subtheme of *External Flaw as a Symbol of One’s Inner Flawed Self* is a novel finding. While it is well accepted that self-esteem is poor in BDD, there has been limited discussion surrounding the idea that the perceived external appearance flaws may be a manifestation of a more global concern regarding their core sense of self. Psychoanalytical theorists have long theorized that in BDD the body part perceived as defective is a symbol of another underlying conflict through a process of displacement (35). Phillips (36), however, notes that such perspectives have no empirical evidence and are difficult to test. Beyond psychoanalytic accounts, Veale (37) asserted that a cognitive behavioral model of BDD must address the role of self-definition and overvalued ideas. Our findings suggest that in BDD the self has become completely entwined with the perceived physical flaw. Thus, psychotherapeutic approaches with BDD clients must endeavor to move beyond targeting of maladaptive behaviors and a focus on appearance concerns to address underlying core beliefs and self-concept.

The final master theme, *intolerance of uncertainty about appearance*, highlights how prominently distress experienced in this population was associated with uncertainty, and participants’ identification of this in the development and maintenance of their BDD symptoms. Indeed, several participants felt that “uncertainty” was the most challenging aspect of their experience with BDD. Intolerance of Uncertainty (IU) has been defined as “an individual’s dispositional incapacity to endure the aversive response triggered by the perceived absence of salient, key, or sufficient information, and sustained by the associated perception of uncertainty” [(38), p. 31]. It has been proposed that Intolerance of Uncertainty (IU) is a transdiagnostic construct playing a role in anxiety, depression, eating disorders and OCD (39–41). IU has been postulated as one of the core 6 dysfunctional beliefs contributing to the development and maintenance of OCD (42). IU tends to be highest in OCD patients with checking compulsions as compared to those with other compulsions such as washing. This is noteworthy given repetitive behaviors in BDD largely revolve around checking the body part of concern.

The notion that compulsive behaviors constitute an ineffective attempt to reduce distress associated with uncertainty has permeated the OCD literature for years, but it has scarcely received mention in the BDD literature (43). The authors are aware of only one study which has addressed IU in BDD, showing that BDD participants have high IU and that IU is associated with poorer functioning (44). Perhaps IU has been overlooked in BDD, as individuals with this condition may not immediately present as uncertain or ambivalent; indeed, they often present with a strong conviction and rigidity surrounding a seemingly unwavering belief that they have a very real and noticeable flawed appearance. However, in the late 19th century Morselli wrote, “the dysmorphic patient in the middle of his daily routines is caught by the doubt of his deformity” (45). Thus, it appears at core of the BDD experience is not a robust negative belief regarding one’s body, but rather an innately unstable and oscillating sense of the body. Further research into the role of IU in BDD is warranted, specifically to explore the potential role this factor may play for the development and maintenance of symptoms. Further, it would be valuable to explore whether IU in BDD is specific to appearance concerns or whether this represents a more generalized distress and intolerance of the unknown.

Connected to experiences of uncertainty, the subtheme of *‘Not Just Right’* revealed participants’ experience of strong internal or body-based sensations of abnormality. Bottesi et al. (46) found that Not Just Right Experiences (NJRE) partially mediated the pathway from IU to checking behaviors in OCD. They proposed that IU was a transdiagnostic construct, whereas NJRE represented an OCD-specific mechanism through which IU functioned to shape compulsions. The current study provides preliminary evidence for NJRE in BDD, suggesting that these experiences are not unique to OCD and warrant investigation in BDD and other OCDR disorders.

Finally, the subtheme, *focus on detail over the whole*, describes processes of “zooming in” and focusing on isolated aspects of appearance over the holistic image. This finding provides qualitative support to both the theory proposed by Veale (8) and previous neuropsychological and neuroimaging findings (47–49) that BDD involves a disposition toward a detail-oriented information processing over holistic right hemispheric information processing. Nonetheless, in the current study ‘focus on detail’ was not limited to visual processing, as participants described this same process with regard to felt impressions and other body based sensory feedback. Future research should explore sensory experiences in BDD more broadly, as the detail-oriented focus in BDD may reflect a broader information processing bias that may not limited to visual mechanisms.

### Clinical implications

The results of this study highlight important considerations for psychologists, mental health clinicians, and other professionals working therapeutically with clients with BDD. Currently Cognitive Behavioral Therapy (CBT) including Exposure and Response Prevention (EPR) is the recommended first line treatment for BDD (36). Clinicians working in the field should explore client’s attitudes toward uncertainty and ambiguity, as well as the function BDD behaviors provide clients as solitary attempts to reduce these symptoms may actually increase distress and feelings of lack of control for this population. The current clinical and research interest in IU as a trait specific and transdiagnostic factor has resulted in the development of CBT programs which specifically target IU. For example, Cognitive Behavioral Therapy Targeting Intolerance of Uncertainty [CBT-IU; (50)] and Making Friends with Uncertainty [Making Friends with Uncertainty; (51)]. The developers of MFWU propose that traditional disorder-specific CBT programs target the ‘threat’ element of disorders, that is the ‘worst case scenarios’ or ‘feared outcome’. Instead, IU specific approaches aim to target the client’s relationship with uncertainty itself (51). This is achieved through providing clients with psychoeducation about IU, developing awareness and attunement to body responses to uncertainty, addressing beliefs about uncertainty (i.e., ‘I cannot handle uncertainty’ and ‘I need to know’), and titrated exposure to uncertainty to build tolerance, acceptance, and safety in the face of the unknown. Clinicians working with BDD clients may which to utilize IU measures to assess and explore the relevance of this factor for the individual. Where clinicians and clients identify IU to play an important role in BDD symptomology it may be beneficial to incorporate IU specific interventions as an add-on to standard BDD protocols. Finally, given the profound feelings of shame among this population and the current study’s finding of ‘*External flaw as a symbol of one’s inner flawed self’* clinicians may also incorporate adjunct interventions, drawing on Compassion Focused Therapy [CFT; (52)] and Internal Family Systems [IFS; (53)].

### Limitations

Our results represent the lived BDD experiences of the 12 individuals interviewed and as with any qualitative research these experiences are not necessarily generalizable. We did include a diverse sample; inclusive of males and females and across a broad age range (19–64 years). It included those who were medicated and unmedicated, as well as those who have undergone cosmetic surgery and those with no such experiences. The group was highly educated, and were treatment-seeking, and these factors might also limit generalizability. Finally, the BDD-YBOCS scores suggest moderately severe symptom severity, and it is therefore possible that individuals with a milder or more severe presentation may have shared different perspectives about their experiences.

## Conclusion

In summary, we describe detailed qualitative accounts validating the seriousness and debilitating nature of BDD, with participants feeling dominated by intrusive thoughts and repetitive behaviors, feeling trapped within their bodies, experiencing a sense of hopelessness and a desire to escape, and experiencing significant functional impairment. They experienced strong feelings of defectiveness and shame, extending beyond appearance, to feelings about their core inner person. A key and novel finding is that these individuals experienced high levels of doubt and uncertainty, which represents a possible developmental or maintenance factor fuelling compulsive checking behaviors in BDD. It is recommended that future research explore the role of shame and intolerance of uncertainty further as these factors may present avenues for innovative interventions for those living with BDD.

## Data availability statement

The raw data supporting the conclusions of this article will be made available by the authors, without undue reservation.

## Ethics statement

The studies involving humans were approved by Swinburne University and St. Vincent’s Hospitals’ Human Research Ethics Committees. The studies were conducted in accordance with the local legislation and institutional requirements. The participants provided their written informed consent to participate in this study. Written informed consent was obtained from the individual(s) for the publication of any potentially identifiable images or data included in this article.

## Author contributions

SB, SR, and DC jointly conceived and planned research study. SB and NT designed qualitative approach including development of interview schedule. SB carried out data collection including interviews and transcription, conducted initial qualitative analysis, and wrote first version of the study for purpose of doctoral thesis. IR and NT provided secondary qualitative analysis resulting in arrival at final themes. SB and SR jointly wrote manuscript for publication. IR, NT, and DC provided writing edits and input to final version of manuscript submitted to this journal. All authors contributed to the article and approved the submitted version.

## References

[1] American Psychiatric Association. Diagnostic and statistical manual of mental disorders. 5th ed. Virginia, US: American Psychiatric Association (2013).

[2] Malcolm A Labuschagne I Castle D Terrett G Rendell PG Rossell SL. The relationship between body dysmorphic disorder and obsessive-compulsive disorder: A systematic review of direct comparative studies. Aust N Z J Psychiatry. (2018) 52:1030–49. 30238784 10.1177/0004867418799925

[3] Rief W Buhlmann U Wilhelm S Borkenhagen A Brähler E. The prevalence of body dysmorphic disorder: A population-based survey. Psychol Med. (2006) 36:877–85. doi: 10.1017/S0033291706007264 16515733

[4] Veale D Gledhill LJ Christodoulou P Hodsoll J. Body dysmorphic disorder in different settings: A systematic review and estimated weighted prevalence. Body Image. (2016) 18:168–86. doi: 10.1016/j.bodyim.2016.07.003 27498379

[5] Buhlmann U Glaesmer H Mewes R Fama JM Wilhelm S Brähler E. Updates on the prevalence of body dysmorphic disorder: a population-based survey. Psychiatry Res. (2010) 178:171–5. doi: 10.1016/j.psychres.2009.05.002 20452057

[6] Phillips K Coles ME Menard W Yen S Fay C Weisberg RB. Suicidal ideation and suicide attempts in body dysmorphic disorder. J Clin Psychiatry. (2005) 66:717–25. doi: 10.4088/JCP.v66n0607 15960564

[7] Phillips K Menard W Quinn E Didie E Stout R. A 4-year prospective observational follow-up study of course and predictors of course in body dysmorphic disorder. Psychol Med. (2013) 43:1109–17. 23171833 10.1017/S0033291712001730

[8] Veale D. Advances in a cognitive behavioural model of body dysmorphic disorder. Body Image. (2004) 1:113–25. doi: 10.1016/S1740-1445(03)00009-3, PMID: 18089145

[9] Veale D Neziroglu F. Body dysmorphic disorder: A treatment manual. Hoboken, NJ: John Wiley & Sons (2010).

[10] Veale D Gournay K Dryden W Boocock A Shah F Willson R. Body dysmorphic disorder: a cognitive behavioural model and pilot randomised controlled trial. Behav Res Ther. (1996) 34:717–29. doi: 10.1016/0005-7967(96)00025-3 8936754

[11] Wilhelm S Phillips KA Fama JM Greenberg JL Steketee G. Modular cognitive–behavioral therapy for body dysmorphic disorder. Behav Ther. (2011) 4:624–33. 10.1016/j.beth.2011.02.002 PMC3320734 22035991

[12] Neziroglu F Khemlani-Patel S Veale D. Social learning theory and cognitive behavioral models of body dysmorphic disorder. Body Image. (2008) 5:28–38. doi: 10.1016/j.bodyim.2008.01.002 18337196

[13] Silver J Reavey P. “He’s a good-looking chap aint he?”: narrative and visualisations of self in body dysmorphic disorder. Soc Sci Med. (2010) 70:1641–7. doi: 10.1016/j.socscimed.2009.11.042, PMID: 20202730

[14] Silver J Reavey P Fineberg N. How do people with body dysmorphic disorder view themselves? A thematic analysis. Int J Psychiatry Clin Pract. (2010) 14:190–7. doi: 10.3109/13651501003735492, PMID: 24917319

[15] Silver J Farrants J. ‘I once stared at myself in the Mirror for eleven hours.’ Exploring mirror gazing in participants with body dysmorphic disorder. J Health Psychol. (2015) 21:2647–57. doi: 10.1177/1359105315581516, PMID: 25947231

[16] Oakes A Collison J Milne-Home J. Repetitive, safe, and automatic: the experience of appearance-related Behaviours in body dysmorphic disorder. Aust Psychol. (2016) 52:12247. doi: 10.1111/ap.12247

[17] Brohede S Wijma B Wijma K Blomberg K. ‘I will be at death’s door and realize that I’ve wasted maybe half of my life on one body part’: the experience of living with body dysmorphic disorder. Int J Psychiatry Clin Pract. (2016) 20:191–8. doi: 10.1080/13651501.2016.1197273, PMID: 27314665

[18] Craythorne SL Shaw RL Larkin M. A phenomenological exploration of self-identified origins and experiences of body dysmorphic disorder. Front Psychol. (2022) 13:963810. doi: 10.3389/fpsyg.2022.963810 36248531 PMC9563380

[19] Smith JA. Evaluating the contribution of interpretative phenomenological analysis. Health Psychol Rev. (2011) 5:9–27. doi: 10.1080/17437199.2010.510659

[20] Smith JA. Beyond the divide between cognition and discourse: using interpretative phenomenological analysis in health psychology. Psychol Health. (1996) 11:261–71. doi: 10.1080/08870449608400256

[21] Smith J Osborn M. Interpretative phenomenological analysis In: Smith JA, editor. Qualitative psychology: A practical guide to research methods. 2nd ed. Los Angeles, CA: Sage (2008). 53–80.

[22] Lecrubier Y Sheehan DV Weiller E Amorim P Bonora I Sheehan KH. The Mini international neuropsychiatric interview (MINI). A short diagnostic structured interview: reliability and validity according to the CIDI. Eur Psychiatry. (1997) 12:224–31. doi: 10.1016/S0924-9338(97)83296-8

[23] Sheehan D Lecrubier Y Sheehan KH Janavs J Weiller E Keskiner A. The validity of the Mini international neuropsychiatric interview (MINI) according to the SCID-P and its reliability. Eur Psychiatry. (1997) 12:232–41. doi: 10.1016/S0924-9338(97)83297-X

[24] Phillips KA. Body dysmorphic disorder diagnostic module. Belmont, MA: McLean Hospital (1994).

[25] Phillips KA Hollander E Rasmussen SA Aronowitz BR. A severity rating scale for body dysmorphic disorder: development, reliability, and validity of a modified version of the Yale-Brown obsessive compulsive scale. Psychopharmacol Bull. (1997) 33:17–22. PMID: 9133747

[26] Eisen JL Phillips KA Baer L Beer DA Atala KD Rasmussen SA. The brown assessment of beliefs scale: reliability and validity. Am J Psychiatr. (1998) 155:102–8. doi: 10.1176/ajp.155.1.102, PMID: 9433346

[27] Lovibond P. Manual for the depression anxiety stress scales. Sydney: The Psychology Foundation of Australia Inc. (1995).

[28] Elliott R Fischer CT Rennie DL. Evolving guidelines for publication of qualitative research studies in psychology and related fields. Br J Clin Psychol. (1999) 38:215–29. doi: 10.1348/014466599162782, PMID: 10532145

[29] Angelakis I Gooding PA Panagioti M. Suicidality in body dysmorphic disorder (BDD): a systematic review with meta-analysis. Clin Psychol Rev. (2016) 49:55–66. doi: 10.1016/j.cpr.2016.08.002, PMID: 27607741

[30] Phillips K Menard W. Suicidality in body dysmorphic disorder: a prospective study. Am J Psychiatr. (2006) 163:1280–2. doi: 10.1176/ajp.2006.163.7.1280, PMID: 16816236 PMC1899233

[31] Veale D Riley S. Mirror, mirror on the wall, who is the ugliest of them all? The psychopathology of mirror gazing in body dysmorphic disorder. Behav Res Ther. (2001) 39:1381–93. doi: 10.1016/S0005-7967(00)00102-9, PMID: 11758697

[32] Windheim K Veale D Anson M. Mirror gazing in body dysmorphic disorder and healthy controls: effects of duration of gazing. Behav Res Ther. (2011) 49:555–64. doi: 10.1016/j.brat.2011.05.003 21726855

[33] Baldock E Anson M Veale D. The stopping criteria for mirror-gazing in body dysmorphic disorder. Br J Clin Psychol. (2012) 51:323–44. doi: 10.1111/j.2044-8260.2012.02032.x 22803938

[34] Abramowitz JS Deacon BJ. Psychometric properties and construct validity of the obsessive-compulsive inventory–revised: replication and extension with a clinical sample. J Anxiety Disord. (2006) 20:1016–35. doi: 10.1016/j.janxdis.2006.03.001, PMID: 16621437

[35] Lemma A. Being seen or being watched? A psychoanalytic perspective on body dysmorphia. Int J Psychoanal. (2009) 90:753–71. doi: 10.1111/j.1745-8315.2009.00158.x, PMID: 19709023

[36] National Institute of Health and Care Excellence. *Obsessive Compulsive Disorder and Body Dysmorphic Disorder: Treatment Clinical Guidelines*. CG31. (2005). Available at: https://www.nice.org.uk/guidance/cg31. 31869034

[37] Veale D. Over-valued ideas: a conceptual analysis. Behav Res Ther. (2002) 40:383–400. doi: 10.1016/S0005-7967(01)00016-X, PMID: 12002896

[38] Carleton RN. Into the unknown: a review and synthesis of contemporary models involving uncertainty. J Anxiety Disord. (2016) 39:30–43. doi: 10.1016/j.janxdis.2016.02.007, PMID: 26945765

[39] Einstein DA. Extension of the transdiagnostic model to focus on intolerance of uncertainty: a review of the literature and implications for treatment. Clin Psychol Sci Pract. (2014) 21:280–300. doi: 10.1111/cpsp.12077, PMID: 25400336 PMC4204511

[40] Kesby A Maguire S Brownlow R Grisham JR. Intolerance of uncertainty in eating disorders: an update on the field. Clin Psychol Rev. (2017) 56:94–105. doi: 10.1016/j.cpr.2017.07.002, PMID: 28710918

[41] Mahoney AE McEvoy PM. A transdiagnostic examination of intolerance of uncertainty across anxiety and depressive disorders. Cogn Behav Ther. (2012) 41:212–22. doi: 10.1080/16506073.2011.622130, PMID: 22032195

[42] Tolin DF Abramowitz JS Brigidi BD Foa EB. Intolerance of uncertainty in obsessive-compulsive disorder. J Anxiety Disord. (2003) 17:233–42. doi: 10.1016/S0887-6185(02)00182-2, PMID: 12614665

[43] Beech H Liddell A. Decision-making, mood states and ritualistic behaviour among obsessional patients. Beech HR, Obsessional states, London: Methuen, pp. 143–160. (1974).

[44] Summers BJ Matheny NL Sarawgi S Cougle JR. Intolerance of uncertainty in body dysmorphic disorder. Body Image. (2016) 16:45–53. doi: 10.1016/j.bodyim.2015.11.002, PMID: 26688272

[45] Morselli E. Sulla dismorfofobia e sulla tafefobia: due forme non per anco descritte di Pizzia con idee fisse. Boll R Accad Genova. (1891) 6:110–9.

[46] Bottesi G Ghisi M Sica C Freeston MH. Intolerance of uncertainty, not just right experiences, and compulsive checking: test of a moderated mediation model on a non-clinical sample. Compr Psychiatry. (2017) 73:111–9. doi: 10.1016/j.comppsych.2016.11.014, PMID: 27939647

[47] Deckersbach T Savage CR Phillips KA Wilhelm S Buhlmann U Rauch SL. Characteristics of memory dysfunction in body dysmorphic disorder. J Int Neuropsychol Soc. (2000):673–81. doi: 10.1017/s1355617700666055 11011514

[48] Feusner JD Townsend J Bystritsky A Bookheimer S. Visual information processing of faces in body dysmorphic disorder. Arch Gen Psychiatry. (2007) 64:1417–25. doi: 10.1001/archpsyc.64.12.1417 18056550

[49] Feusner JD Moody T Hembacher E Townsend J McKinley M Moller H. (2010). Abnormalities of visual processing and frontostriatal systems in body dysmorphic disorder. Arch Gen Psychiatry. (2010):197–205. doi: 10.1001/archgenpsychiatry.2009.190 PMC2853756 20124119

[50] Dugas MJ Ladouceur R. Treatment of GAD: targeting intolerance of uncertainty in two types of worry. Behav Modif. (2000) 24:635–57. doi: 10.1177/0145445500245002, PMID: 11036732

[51] Mofrad L Tiplady A Payne D Freeston M. Making friends with uncertainty: experiences of developing a transdiagnostic group intervention targeting intolerance of uncertainty in IAPT. Feasibility, acceptability and implications. Cogn Behav Therap. (2020) 13:E49. doi: 10.1017/S1754470X20000495

[52] Gilbert P. Compassion focused therapy. Abingdon, Oxon: Routledge (2010).

[53] Schwartz RC. Internal family systems therapy. New York: Guilford Press (1995).

